# The diagnostic value of a breast cancer diagnosis model based on serum MiRNAs and serum tumor markers

**DOI:** 10.1186/s12957-025-03719-z

**Published:** 2025-03-29

**Authors:** Xiaohui Li, Feng Wang, Faquan Lin, Binbin Xie, Yi Liu, Yi Xiao, Kai Qin, Weicheng Li, Qiyan Zeng

**Affiliations:** 1 Department of Biochemistry and Molecular Biology, Guangxi Medical University, Nanning, Guangxi People’s Republic of China; 2 Key Laboratory of Biological Molecular Medicine Research, Education Department of Guangxi Zhuang Autonomous Region, Nanning, Guangxi People’s Republic of China; 3 Department of Clinical Laboratory, First Affiliated Hospital of Guangxi Medical University, Nanning, Guangxi People’s Republic of China

**Keywords:** Hsa-miR-548ao-5p, Hsa-miR-4804-3p, Breast cancer, Tumor markers, Diagnosis

## Abstract

**Background:**

Breast cancer (BCa) is the leading cause of cancer-related death among women worldwide. MicroRNAs (miRNAs) are promising tools for diagnosis and prognosis. This study investigated the role of serum miRNAs and tumor markers (TMs) in the diagnosis of BCa.

**Methods:**

Differentially expressed miRNAs were screened from serum samples of BCa patients and healthy individuals via high-throughput sequencing. The expression of hsa-miR-1911-3p, hsa-miR-4694-5p, hsa-miR-548ao-5p, and hsa-miR-4804-3p in 169 BCa patients and 116 healthy controls was detected via qRT-PCR. Serum tumor-associated antigens were detected by chemiluminescence. Logistic regression was subsequently used to develop the miRNA panel I, TM panel II, and (miRNA + TM) panel III models. Receiver operating characteristic (ROC) curve, precision-recall (PR) curve and decision curve analyses (DCA) were performed to assess the accuracy of the three models for BCa diagnosis. Additionally, the relationships between miRNA expression and the clinical characteristics of patients with BCa were assessed.

**Results:**

Four serum miRNAs (hsa-miR-1911-3p, hsa-miR-548ao-5p, hsa-miR-4694-5p, and hsa-miR-4804-3p) were newly associated with BCa. The miRNA panel I based on hsa-miR-548ao-5p and hsa-miR-4804-3p showed greater diagnostic effectiveness for BCa than TM panel II based on cancer antigen 125 (CA125) and cancer antigen 153 (CA153), with AUC values of 0.816 and 0.777, respectively. (miRNA + TM) panel III had higher diagnostic effectiveness than miRNA panel I, with an AUC value of 0.870. The expression of miR-548ao-5p and miR-4804-3p is closely related to clinical features, such as human epidermal growth factor receptor 2 (HER2), estrogen receptor (ER), progesterone receptor (PR), HER2-enriched subtype, stage III/IV, and lymph node-transplanted breast cancer.

**Conclusion:**

MiR-548ao-5p and miR-4804-3 could serve as potential biomarkers for the diagnosis of BCa.

**Supplementary Information:**

The online version contains supplementary material available at 10.1186/s12957-025-03719-z.

## Introduction

Breast cancer (BCa) is a complex and highly heterogeneous malignant tumor influenced by genetic susceptibility, environmental factors, and lifestyle factors [1]. Each patient exhibits varying biological behaviors, performances, and prognoses. As of 2022, BCa ranks as the second leading cause of cancer incidence worldwide and the primary cause of female cancer death [2]. Early diagnosis of BCa can significantly improve long-term survival and provide a good prognosis for patients [3, 4]. Current medical systems are dedicated to reducing BCa mortality through prevention, awareness-raising, early detection, and treatment [5].

Current clinical screening and diagnostic methods for BCa encompass a range of imaging techniques, including X-ray mammography, ultrasound, and magnetic resonance imaging, as well as pathological examinations, such as biopsy and tissue examination [6–8]. However, the former is associated with high costs and radiation exposure, whereas the latter carries a significant risk owing to its invasive nature. Each testing method varies in terms of practicality, sensitivity, and specificity. Conversely, blood tests are widely accepted because of their low cost and noninvasive nature. Cancer antigen 153 (CA153) is a substance that stimulates the body’s defense system and plays a crucial role in the diagnosis and prognosis of BCa [9, 10]. Nonetheless, CA153 exhibits low sensitivity and is susceptible to producing false negative results. So, identifying novel and effective biomarkers to enhance early detection of BCa remains one of the most significant challenges in the field of oncology.

MicroRNAs (miRNAs) are small, noncoding endogenous RNA molecules that serve as epigenetic regulators by binding to the 3’UTR of the target messenger RNA to modulate target gene expression [11, 12]. MiRNAs play pivotal roles in regulating intricate genetic networks, including cell proliferation, apoptosis, therapeutic effects, and disease development [13, 14]. Recent research has demonstrated a significant reduction in miR-328-3p in metastatic BCa [15]. This reduction has been found to promote the growth and metastasis of BCa through the miRNA-328-3p-carnitine palmitoyl transferase 1a pathway. A separate study demonstrated that miR-128 and miR-223 can mitigate Tamoxifen resistance by reducing excessive cholesterol consumption [16]. Xu et al. found that Lin28A-miRNA proteolysis-targeting chimeras effectively inhibited the proliferation and migration of BCa cells [17]. Furthermore, when combined with tamoxifen, it exhibited significant tumor-suppressive effects in breast cancer mouse model. Numerous experiments have demonstrated that the enrichment or depletion of specific miRNA variants may be correlated with tumor invasiveness, drug resistance, and clinical prognosis [18]. As a result, miRNA biomarkers can serve as adjunctive diagnostic tools for BCa and can be analyzed at various stages of disease progression.

This study aimed to identify differentially expressed serum miRNAs, including hsa-miR-1911-3p, hsa-miR-4694-5p, hsa-miR-548ao-5p, and hsa–miR-4804-3p, in BCa. These miRNAs are combined with commonly used serum protein markers for tumors such as CA153, cancer antigen 125 (CA125), carcinoembryonic Antigen (CEA), cancer antigen 199 (CA199), and alpha-fetoprotein (AFP). The goal is to develop a high-sensitivity, high-specificity, and high-accuracy risk assessment model to improve the diagnosis and treatment of BCa.

## Materials and methods

### Experimental design

Our research was conducted in three phase. In the discovery phase, high-throughput sequencing was used to screen differentially expressed miRNAs in serum samples from both BCa patients and healthy individuals. The training phase involved the detection of candidate miRNAs and serum tumor markers (including CEA, CA-152, CA-125, CA-199, and AFP) via qRT-PCR and chemiluminescence in 116 BCa patients and 68 healthy serum samples. Subsequently, multivariable logistic regression methods were utilized to develop diagnostic models for BCa based on serum miRNAs (referred to as miRNA panel I), serum tumor markers (referred to as TM panel II), and a combination of miRNAs with serum tumor markers (referred to as (miRNA + TM) panel III). The diagnostic performance of the three models was assessed via receiver operating characteristic (ROC) curve. The performance of the candidate miRNAs was subsequently revalidated in a separate validation phase involving 53 BCa patients and 46 healthy individuals. The cut-off value determined during the training phase was applied to the validation phase to evaluate the predictive accuracy of the three molecular signatures. The ROC curve, precision-recall (PR) curve, and decision curve analysis (DCA) were used to evaluate the diagnostic efficiency and clinical applicability of the three models. We investigated the correlation between the expression of miR-548ao-5p or miR-4804-3p and the clinical characteristics of patients with BCa. Furthermore, serum samples from patients with hepatocellular carcinoma, lung cancer, and gastric cancer were also collected for analysis to confirm the specificity of the candidate miRNAs.

### Study objects

The inclusion criteria for breast cancer patients in this study were as follows: non-pregnant women aged over 18 years who had provided informed consent and received a diagnosis of breast cancer confirmed by pathological examination. Additionally, individuals with other types of tumors, those suffering from severe cardiopulmonary diseases, liver or kidney insufficiency, or those exhibiting significant coagulation disorders were excluded from the study. The study involved 169 female patients who were clinically diagnosed with BCa at the First Affiliated Hospital of Guangxi Medical University between June 2020 and July 2021. A comparison group of 114 healthy individuals during the same time period was also included. In addition, we obtained 40 samples of BCa tumor tissue and adjacent tumor tissue that had undergone surgical resection from patients treated at Guangxi Medical University Cancer Hospital. Demographic characteristics and medical history information were also collected.

### Sample preparation

Blood samples should be collected within 2 h, and miRNA should be isolated from 200 µL of serum samples via the miRNeasy Serum/Plasma Kit (Qiagen, Germany) following the manufacturer’s instructions. The extracted miRNA was then dissolved in 14 µL of ultrapure water. In contrast, the collected tumor tissues were promptly submerged in RNA protective solution (Omega Bio-Tek, USA). Subsequently, 20–30 mg of tissue was homogenized via RIPA lysis buffer (Amresco, USA) for total RNA extraction with total DNA/RNA isolation kit (Omega Bio-Tek, USA). The entire process was carried out at low temperatures.

### Screening and validation of differential serum MiRNA expression in BCa patients

Candidate miRNAs were identified using Illumina HiSeq SE50 sequencing, with detailed procedures in the supplementary methods. The total miRNA of 2 µg was reverse-transcribed into cDNA using the tailing method, in accordance with the instructions provided by the Mir-X miRNA First-Strand Synthesis Kit (Takara, Japan). qRT-PCR was performed with the the TB Green Premix Ex Taq II Kit (Takara, Japan) and miRNA-specific primers (GeneCopoeia, USA) on the LightCycler 96 PCR platform. Two-step qRT-PCR was employed for amplification. The reaction conditions were as follows: pre-denaturation at 95℃ for 30s, followed by one cycle; then amplification at 95℃ for 5s and at 60℃ for 30s, repeated for 40 cycles. The solution curve was established as the temperature gradually increased from 65℃ to 95℃, while the variation in fluorescence intensity was continuously monitored. All reactions were conducted in triplicate. A total of 5 healthy individuals and 5 breast cancer patients were randomly selected to evaluate the expression levels of U6, hsa-miR-16-5p, and the spike-in control cel-miR-39. This approach aimed to identify suitable reference genes. Finally, relative expression was calculated using the 2^−△△Ct^ method. The primers sequences were shown in Table 1.

**Table 1:** Primer sequences for qRT-PCR

Variable	Primer sequences
MiR-1911-3p Forward Primer	5’-AGGCATTGTGGTCTCCAAAA-3’
MiR-548ao-5p Forward Primer	5’-GAAGTAACTACGCTTTTTGCA-3’
MiR-4694-5p Forward Primer	5’-GGTGTTATCCTATCCATTTGCAAA-3’
MiR-4804-3p Forward Primer	5’-CTTAACCTTGCCCTCGAAAAA-3’
U6 Forward Primer	5’-GGAACGATACAGAGAAGATTAGC-3’
U6 Reverse Primer	5’-TGGAACGCTTCACGAATTTGCG-3’

### Detection of serum markers

Using acridinium ester as the light-emitting substrate, we measured CA153, CA152, CA199, CEA, and AFP using direct chemiluminescence with a Abbott ARCHITECT c8000 instrument. Specifically, the two-point immunization method is utilized. The antigen in the sample is bound to a monoclonal antibody in the solid phase and then to a polyclonal antibody labeled with acridinium ester in the liquid phase. The resulting acridinium ester subsequently undergoes a chemical reaction in an alkaline environment to generate a specific light signal for quantifying the concentration of the antigen.

### Development of a diagnostic model for breast cancer and evaluation of its diagnostic efficacy

During the training phase, statistically significant indicators from the univariate analysis were included in a binary logistic regression to develop a diagnostic model for BCa, using the presence of BCa as the outcome variable. Each independent logistic regression model contained no more than four variables, adhering to the minimum requirement of 10 Events Per Variable, resulting in high stability [19, 20]. The weight of each index was determined through logistic regression analysis, and individual risk scores were calculated. Risk score distribution across subjects was visualized using waterfall plots. ROC and AUC analyses illustrated cut-off points between sensitivity and specificity at various diagnostic thresholds. During the training phase, the optimal cut-off value, sensitivity, and specificity were determined on the basis of Youden’s index (Youden’s inde = sensitivity + specificity − 1). A test result is considered negative and indicative of a healthy individual when the indicator falls below the cut-off value. Conversely, a result above the cut-off value indicates positivity and suggests the presence of BCa. Cut-off values from the training phase were applied to other phases to calculate their respective sensitivity, specificity, and accuracy. Finally, the diagnostic and clinical efficacy of the three models was assessed using ROC curves, PR curves, and DCA.

### Statistical analysis

All the data were processed via SPSS 25.0, GraphPad Prism 8, R software and Python. The Kolmogorov-Smirnov test was used to determine the data distribution for each group. Continuous variables with a normal distribution were assessed by Student’s t-test or ANOVA and are presented as the mean ± standard deviation (SD), whereas nonnormally distributed data were evaluated using the Mann-Whitney U test or Kruskal-Wallis H tests and represented as the median and interquartile range (IQR). Categorical variables were analyzed via Pearson’s chi-square test or Fisher’s exact test. All the statistical analyses employed two-tailed tests, with significance set at *P* < 0.05.

## Results

### Clinical characteristics

In this study, 169 serum samples were collected from patients with BCa, with an average age of 47 years. Simultaneously, 114 serum samples from healthy individuals, with an average age of 46 years, were collected for comparison. The BCa patients and healthy individuals were randomly divided into two phases: the training phase and the validation phase. Table 2 indicates that there were no significant differences in molecular subtypes or TNM anatomic staging between the training and validation phases (all *p* > 0.05).

**Table 2:** Clinical characteristics of all BCa patients included in the study

Variable	Training phase		Validation phase		*P* value
	BCa (*N* = 116)	*N* (*N* = 68)	BCa (*N* = 53)	*N* (*N* = 46)	
Age	46 (38–53)	47(36–54)	49 (42–56)	44(36–54)	0.358
Molecular Subtype					0.575
Luminal A	25 (24.0%)		9 (17.6%)		
Luminal B	42 (40.4%)		22 (43.2%)		
HER2-enriched	21 (20.2%)		11 (21.6%)		
TNBC	16 (15.4%)		9 (17.6%)		
ER status					0.485
Negative	38 (36.5%)		22 (42.3%)		
Positive	66 (63.5%)		30 (57.7%)		
PR status					0.534
Negative	51 (48.6%)		28 (53.8%)		
Positive	54 (51.4%)		24 (46.2%		
HER2 status					0.870
Negative	58 (56.3%)		30 (57.7%)		
Positive	45 (43.7%)		22 (42.3%)		
TNM Classification					0.450
Stage I	28 (24.3%)		7 (15.2%)		
Stage II	39 (33.9%)		14 (30.4%)		
Stage III	23 (20.0%)		11 (24.0%)		
Stage IV	25 (21.8%)		14 (30.4%)		
T					0.304
T1	34 (31.8%)		8 (20.5%)		
T2	52 (48.6%)		18 (46.1%)		
T3	14 (13.1%)		9 (23.1%)		
T4	7 (6.5%)		4 (10.3%)		
Lymph node metastasis					0.680
No	54 (50%)		18 (46.2%)		
Yes	54 (50%)		21 (53.8%)		
Distal metastasis					0.257
No	89 (78.1%)		32 (69.6%)		
Yes	25 (21.9%)		14 (30.4%)		

Abbreviations: HER2, human epidermal growth factor receptor 2; TNBC, Triple-Negative Breast Cancer; ER, estrogen receptor; PR, progesterone receptor; TNM, Tumor-Node-Metastasis. T1: Tumor diameter ≤ 20 mm; T2: 20 mm < tumor diameter ≤ 50 mm; T3: Tumor diameter > 50 mm; T4: Any tumor size with chest wall or skin invasion. Age was assessed using the Mann-Whitney U test. T stage was evaluated through Fisher’s exact test, and all other variables were analyzed with the Pearson’s chi-square test

### Differential expression of serum MiRNAs in patients with breast cancer

**Fig. 1:**
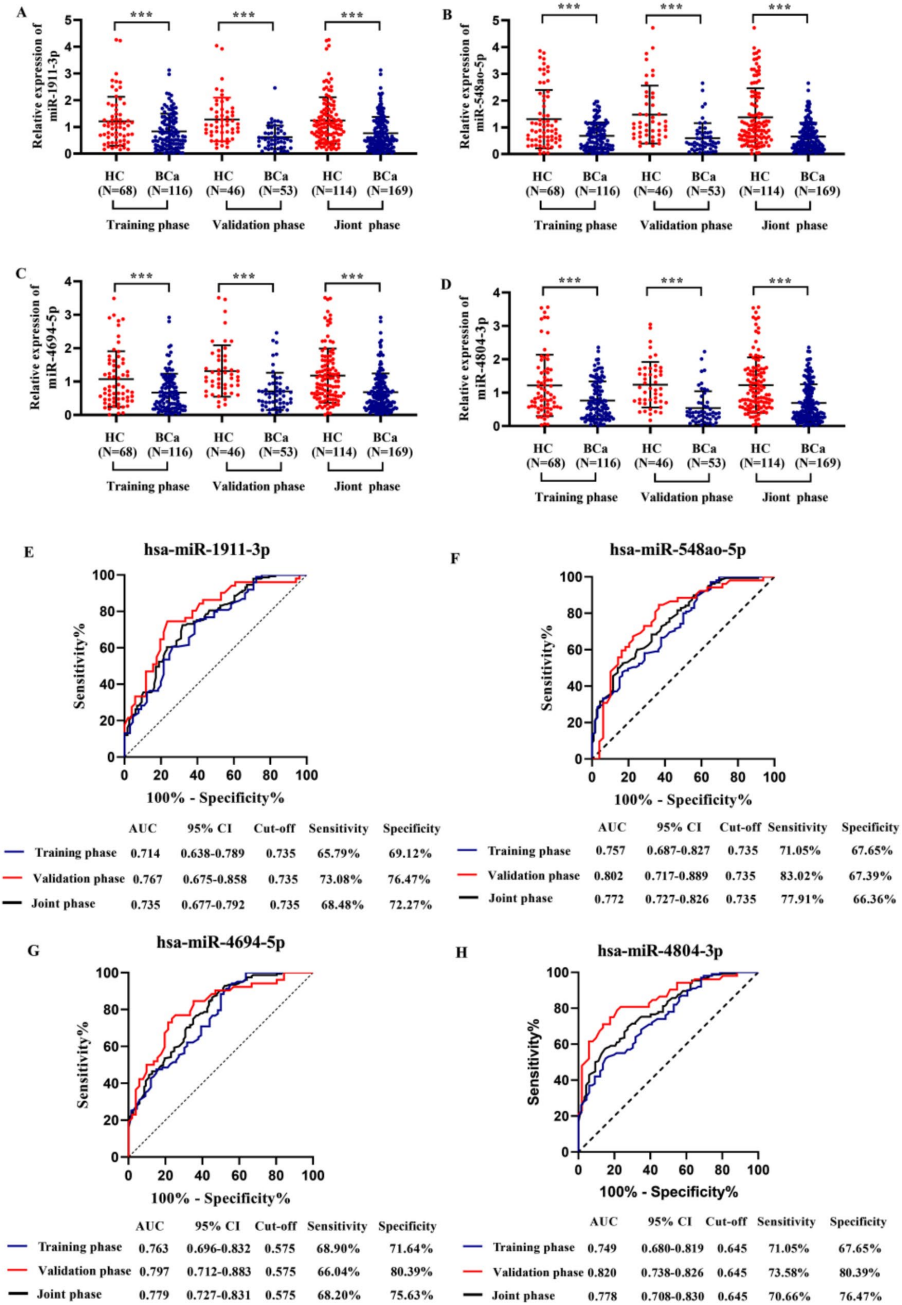
The levels of miR-1911-3p, miR-548ao-5p, miR-4694-5p, and miR-4804-3p were evaluated in the training phase, validation phase, and joint phase. Relative expression levels of (**A**) miR-1911-3p, (**B**) miR-548ao-5p, (**C**) miR-4694-5p, and (**D**) miR-4804-3p. ROC curve showing the diagnostic value of miR-1911-3p (**E**), miR-548ao-5p (**F**), miR-4694-5p (**G**), and miR-4804-3p (**H**) in BCa. HC: Healthy control; BCa: Breast cancer; *** *P* < 0.001

Initial screening on the Illumina HiSeq SE50 platform identified 123 differentially expressed miRNAs between BCa patients and healthy controls (data not shown). Currently, there is no literature linking the four differentially expressed miRNAs (including hsa-miR-1911-3p, hsa-miR-548ao-5p, hsa-miR-4694-5p, and hsa-miR-4804-3p) with BCa. In this study, U6 was chosen as the internal reference gene for qRT-PCR due to its stability. For detailed results, please refer to supplementary table S1. During training, expression levels of these four serum candidate miRNAs in 116 BCa patients were significantly lower than those in healthy individuals (Fig. 1A-D; *P* < 0.001). ROC analysis revealed that the candidate miRNAs had diagnostic potential for BCa, with AUCs ranging from 0.714 to 0.763 (Fig. 1E-H, *P* < 0.05). In a validation phase involving 46 BCa patients and 53 healthy controls, their expression patterns remained consistent, yielding AUCs from 0.767 to 0.820, thus confirming their diagnostic efficacy (Fig. 1E-H, *P* < 0.05).

### Development and validation of a 2-miRNA panel for predicting the occurrence of breast cancer

During the training phase, a logical analysis revealed that miR-548ao-5p and miR-4804-3p were identified as independent predictors for inhibiting the development of BCa, whereas miR-1911-3p and miR-4694-5p did not have independent predictive value. As a result, a model known as miRNA panel I was developed to assess individual BCa risk. The formula for determining the risk score in miRNA panel I is as follows (Supplementary table S2):

Risk score = 2.494 + (miR-548ao-5p × (-1.568)) + (miR-4804-3p × (-1.134)).

**Fig. 2:**
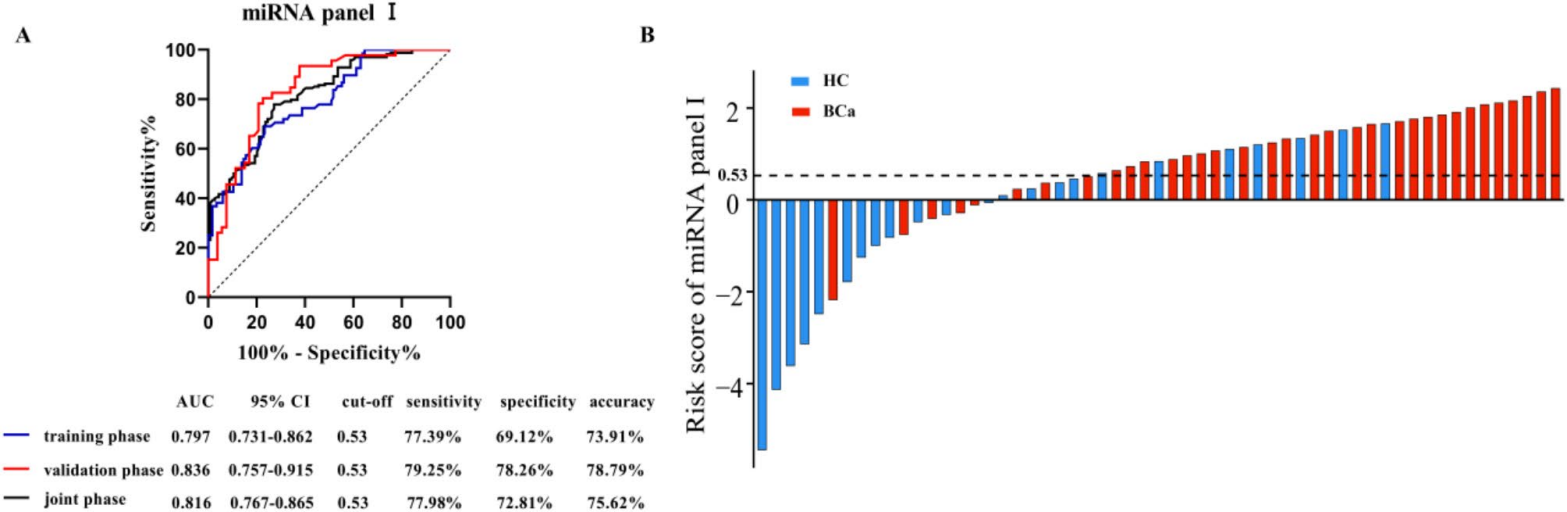
Diagnostic performance of miRNA panel I. (**A**) ROC showing the diagnostic value of miRNA panel I in BCa patients in the training phase, validation phase, and joint phase. (**B**) Waterfall plots illustrating the dispersion of risk scores for each individual. The study included a total of 169 BCa patients and 114 healthy controls. Given the substantial number of specimens, presenting all results in full would be excessively lengthy; therefore, we will aggregate the averages of five adjacent results from each group to create a waterfall plot

This formula was applied at each phase to calculate the individual risk scores. The results revealed that BCa patients tended to have higher risk scores than healthy controls did, with AUCs for the 2-miRNA panel I in the training, validation, and joint phases of 0.797, 0.836 and 0.816, respectively (Fig. 2A-B, *P* < 0.05).

### Performances of CEA, CA125, CA153, CA199 and AFP in breast cancer patients

Monofactor analysis revealed that the serum levels of the tumor markers CEA, CA125, and CA153 were significantly greater in patients with BCa than in healthy controls (Supplementary figure S1, *P* < 0.05). In contrast, the expression of AFP and CA199 did not significantly differ. During the training phase, CEA, CA125, and CA153 were included in a logistic regression equation to develop a diagnostic model for BCa known as TM panel II. The findings revealed that CEA did not emerge as an independent factor in BCa events, whereas CA125 and CA153 were identified as independent contributors. This led to the formulation of the following equation (Supplementary table S3):

TM panel II risk score = (0.15 × CA125) + (0.101 × CA153) - 2.70.

**Fig. 3:**
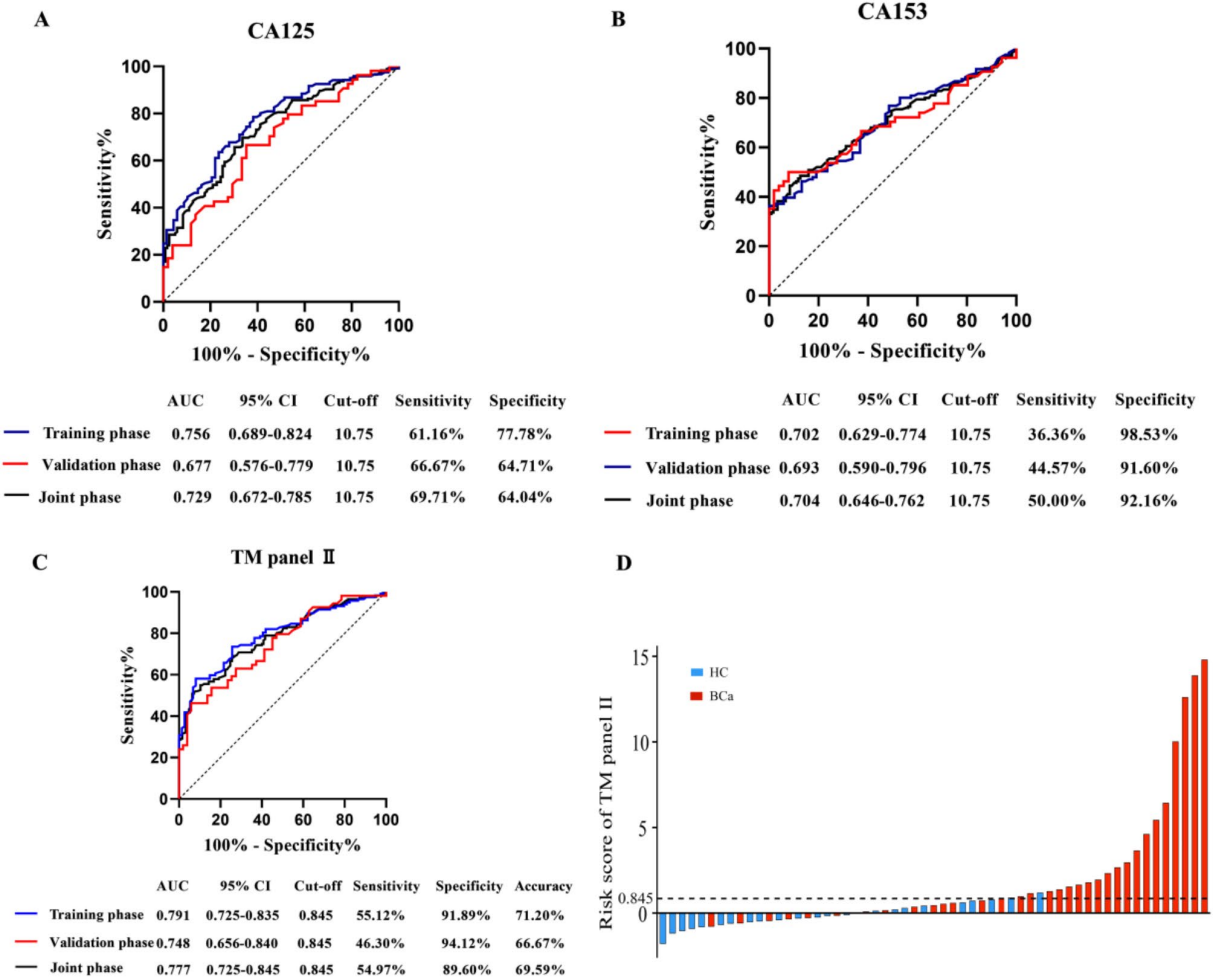
CA125 (**A**), CA153 (**B**), and TM panel II (**C**) demonstrating diagnostic efficacy for BCa in the training phase, validation phase, and joint phase, respectively. (**D**) Waterfall plots depicting the distribution of risk scores for each individual patient. The study included a total of 169 BCa patients and 114 healthy controls. Given the substantial number of specimens, presenting all results in full would be excessively lengthy; therefore, we will aggregate the averages of five adjacent results from each group to create a waterfall plot

ROC analysis showed that the TM Panel II model had better diagnostic value for breast cancer adenocarcinoma than CA125 or CA153 (Fig. 3A-C). Compared with healthy controls, BCa patients exhibited higher risk scores on the TM Panel II (Fig. 3D, *P* < 0.05). This study indicates that serum markers have higher specificity but lower sensitivity for BCa. MiRNA panel I significantly improved the diagnostic sensitivity for BCa, outperforming CA125 and CA153 in terms of diagnostic effectiveness (*P* < 0.05). Moreover, miRNA panel I demonstrated greater accuracy and sensitivity in diagnosing BCa than did TM panel II (*P* < 0.05), thus reducing the likelihood of missed diagnoses in patients.

### Diagnostic value of a combination of MiRNAs with serum tumor markers in breast cancer treatments

To enhance the sensitivity of serum tumor markers in diagnosing BCa, we performed logistic regression analysis during the training phase to combine miR-548ao-5p, miR-4804-3p, CA125, and CA153. This resulted in a new model called (miRNA + TM) panel III. The formula for distinguishing BCa is as follows (Supplementary table S4):

(miRNA + TM) panel III risk score = CA125 × 0.123 + CA153 × 0.069 + miR-548ao × (-1.173) + miR-4804 × (-1.185).

**Fig. 4:**
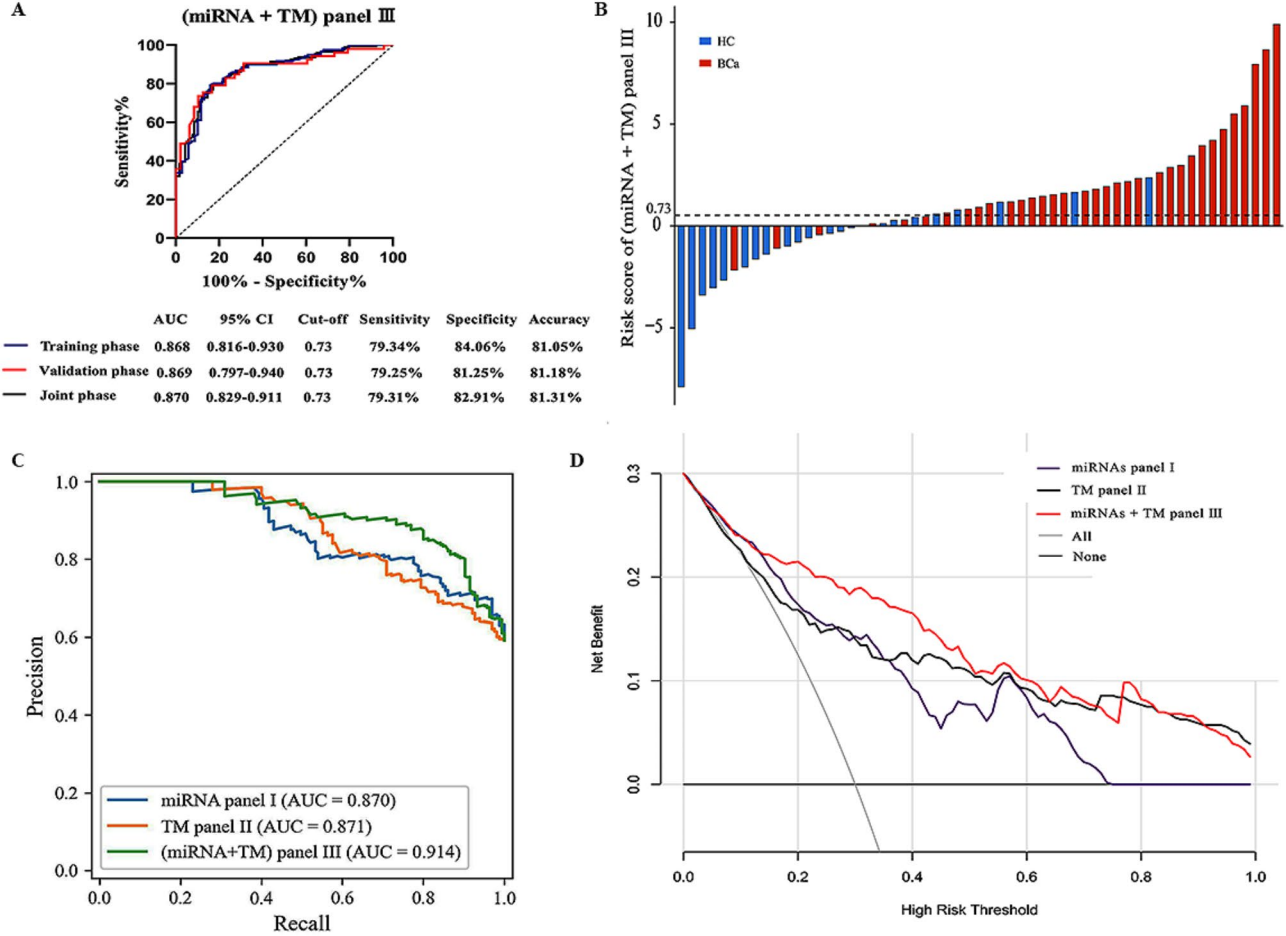
(**A**) ROC curve showing the diagnostic value of (miRNA + TM) panel III in BCa patients in the training, validation, and joint phases.(**B**) Waterfall plots depicting the distribution of risk scores for each individual patient. The study included a total of 169 BCa patients and 114 healthy controls. Given the substantial number of specimens, presenting all results in full would be excessively lengthy; therefore, we will aggregate the averages of five adjacent results from each group to create a waterfall plot. (**C**) PR curve illustrating the precision and recall metrics of the three models. (**D**) DCA demonstrating the clinical advantages of the three models

The ROC curve results revealed that the AUC and accuracy of the diagnostic values in (miRNA + TM) panel III during training, validation, and joint phases were as follows: 0.868 and 81.05%, 0.869 and 81.18%, and 0.870 and 81.31%, respectively (Fig. 4A). BCa patients generally presented higher risk scores on the (miRNA + TM) panel III compared to healthy controls (Fig. 4B, *P* < 0.05). In terms of the area under the precision-recall curve, the (miRNA + TM) panel III demonstrates a value of 0.914, indicating a strong balance between precision and recall for BCa prediction (Fig. 4C). DCA revealed substantial clinical net benefits from the three models. When medical interventions are initiated at identical threshold probabilities, using the (miRNA + TM) Panel III model provides the greatest net benefit for BCa patients (Fig. 4D). Our comparative analyses confirm that the (miRNA + TM) panel III exhibits exceptional sensitivity, specificity, accuracy, and stability. Moreover, it demonstrates even greater efficacy in differentiating BCa patients from healthy individuals.

### Correlations between serum MiRNA expression and the clinical characteristics of patients with breast cancer

The correlations between the expression of miR-548ao-5p or miR-4804-3p and clinical characteristics are outlined in Supplementary table S5.

**Fig. 5:**
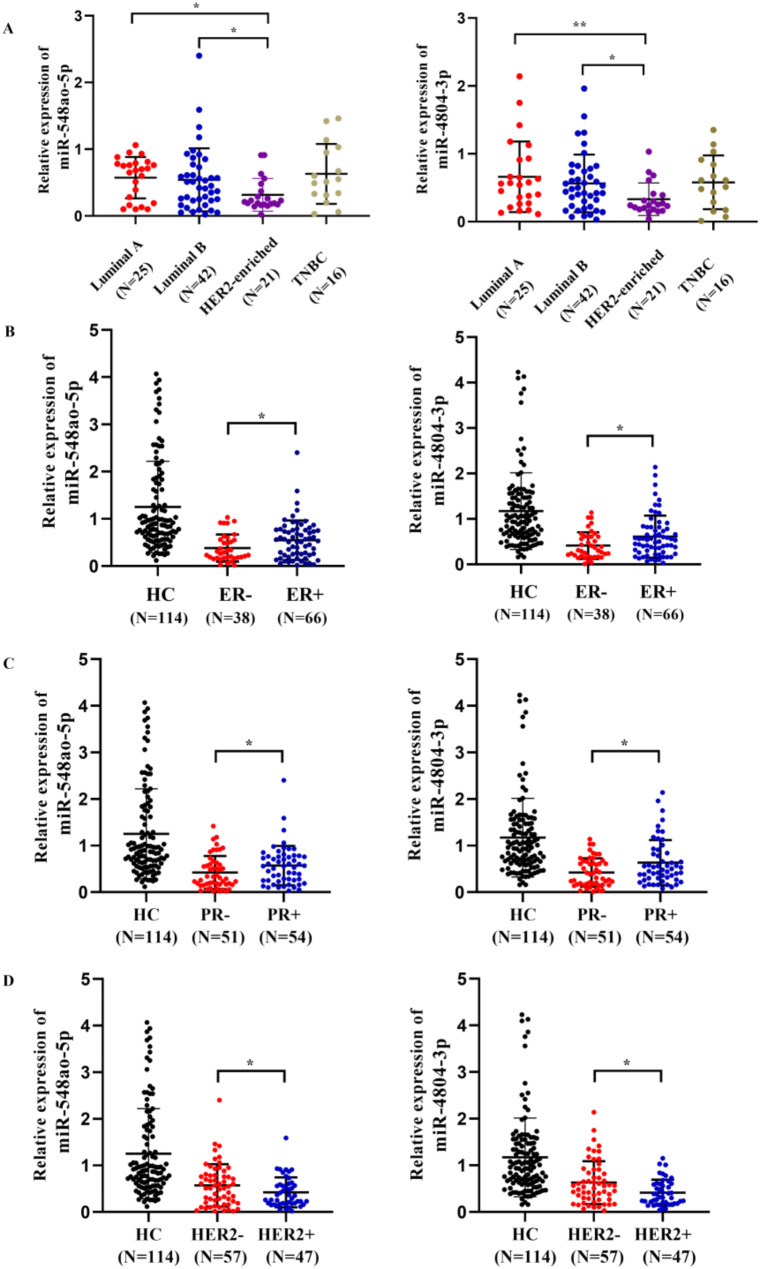
(**A**-**B**) Expression of miR-548ao-3p and miR-4804-3p in different molecular subtypes. (**B**-**D**) The expression of miR-548ao-3p and miR-4804-3p was significantly lower in patients in the ER-, PR-, and HER2 + groups. * *P* < 0.05; ** *P* < 0.01; *** *P* < 0.001

**Fig. 6:**
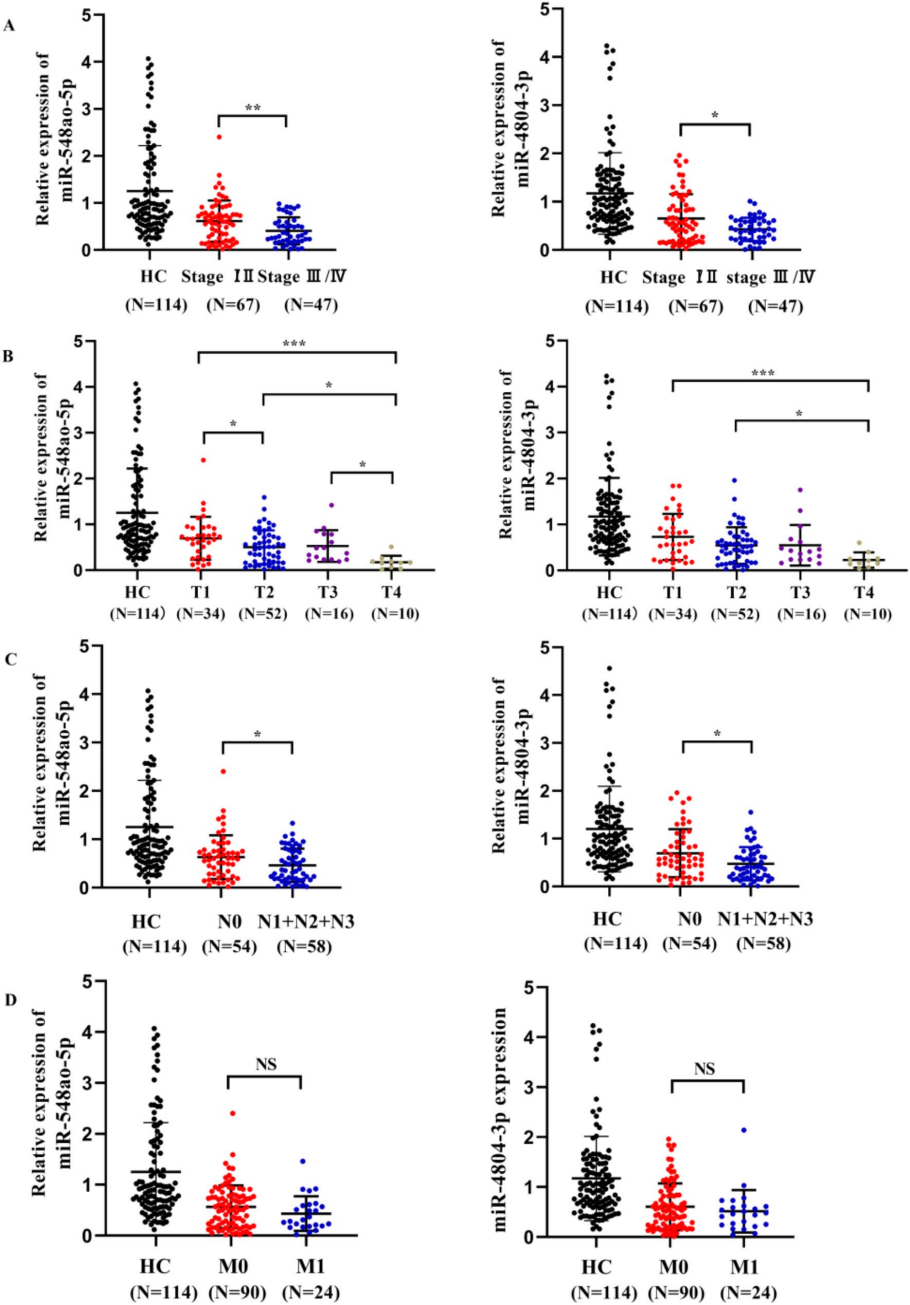
(**A**) The expression of miR-548ao-3p and miR-4804-3p in different TNM anatomic stages of BCa. (**B**-**D**) The expression of miR-548ao-3p and miR-4804-3p was analyzed in relation to the T phase, N phase, and M phase, respectively. NS: Not Significant. * *P* < 0.05; ** *P* < 0.01; *** *P* < 0.001

In the aggregate sample, miR-548ao-5p and miR-4804-3p were expressed at significantly lower levels in the human epidermal growth factor receptor 2 (HER2) enriched subtype (*P* < 0.05, Fig. 5). The expression of miR-548ao-5p and miR-4804-3p was notably lower in patients who were estrogen receptor (ER) negative, progesterone receptor (PR) negative, HER2 positive, lymphatic metastasis and stage III/IV than in those with ER+, PR+, HER2-, no lymphatic metastasis and stage I/II disease (*P* < 0.05, Figs. 5 and 6). Furthermore, there was a noticeable trend toward decreased serum expression levels of miR-548ao-5p and miR-4804-3p as the T phase advanced. To eliminate the influence of confounding factors and assess the reliability of miR-548ao-5p and miR-4804-3p as independent predictors, we incorporated indicators with significant correlations (including ER status, PR status, and HER2 status) into multiple linear regression analyses using miR-548ao-5p and miR-4804-3p as dependent variables, respectively. The results indicated that the HER2 status significantly influences the expression levels of both miR-548ao-5p and miR-4804-3p (Supplementary table S6 and Supplementary table S7). At the same time, after eliminating the mixing factor, the expression level of those miRNAs in HER2 + patients was still significantly lower than that in HER2 − group. Collectively, these findings suggest a potential close association between miR-548ao‐5p and miR‐4804‐3p and the progression of BCa.

### Expression of miR-548ao-3p and miR-4804-3p in breast cancer tissues and in the serum of other types of cancer

**Fig. 7:**
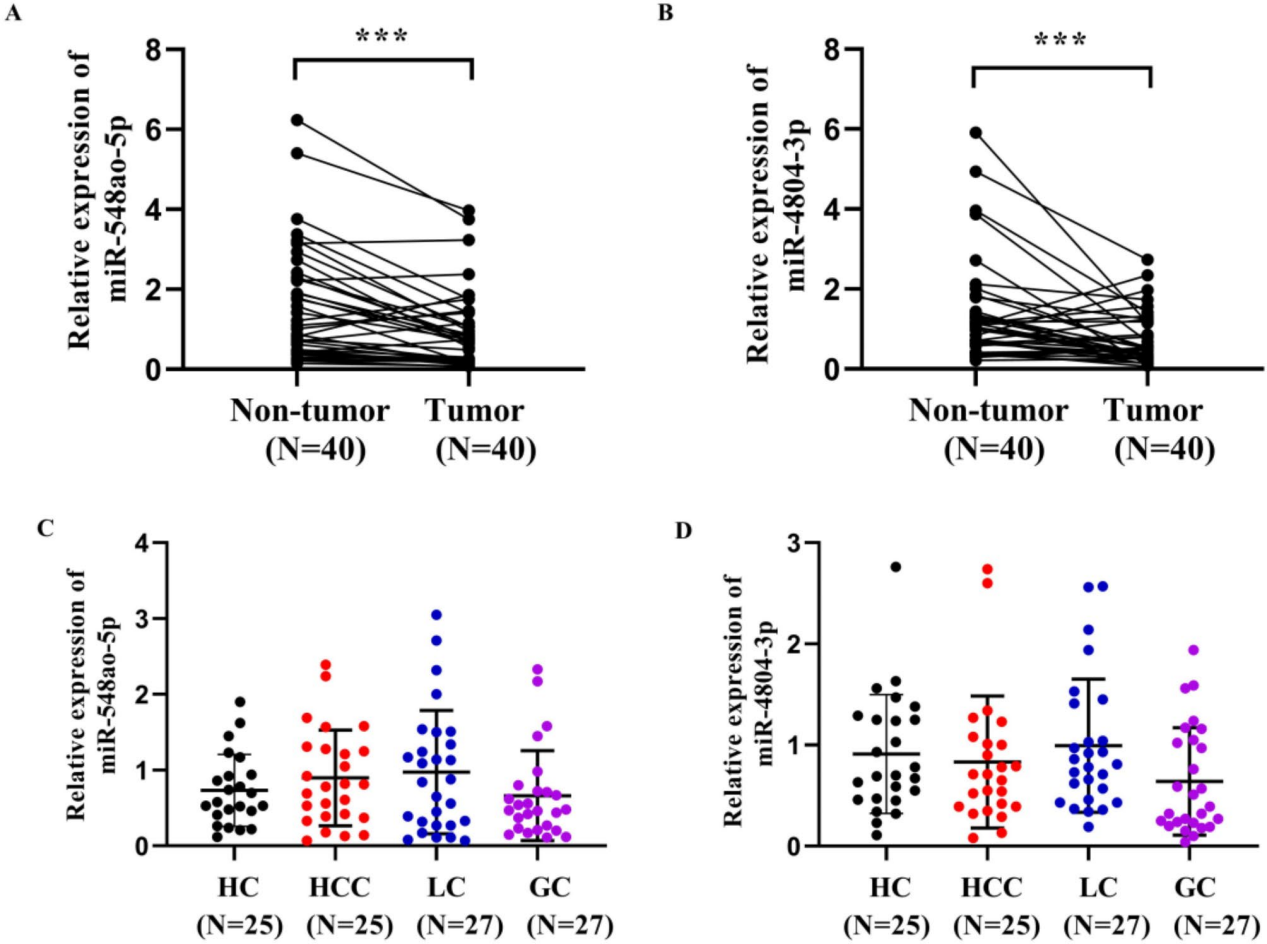
(**A**-**B**) The expression of miR-548ao-5p and miR-4804-3p in breast cancer tumor tissues and nontumor adjacent tissues; (**C**-**D**) The expression of miR-548ao-5p and miR-4804-3p in the serum of hepatocellular carcinoma (HCC), lung cancer (LC), gastric cancer (GC) patients and healthy controls (HC). *** *P* < 0.001

qRT-PCR analysis revealed that the expression of miR-548ao-5p and miR-4804-3p in BCa tumor tissues was significantly lower than that in nontumor adjacent tissues. This finding is consistent with previous observations of significantly lower expression levels of miR-548ao-5p and miR-4804-3p in the serum of BCa patients(Fig. 7A-B, *P* < 0.05). However, the expressions levels of miR-548ao-5p and miR-4804-3p in the hepatocellular carcinoma, lung cancer, and gastric cancer groups did not significantly differ from those in the healthy control group (Fig. 7C-D, *P* > 0.05). This study suggested that the expression patterns of miR-548ao‐5p and miR‐4804‐3p differ between these three cancers and breast cancer, indicating that a risk model composed of these two miRNAs may have specific utility in diagnosing breast cancer.

## Disscious

MiRNAs regulate gene expression by interacting with target mRNA, playing key roles in the cell cycle, development, apoptosis, and disease progression [21, 22]. A recent study achieved selective drug delivery by co-loading miR-34a and photosensitizers within cationic liposomes [23]. This strategy leads to the suppression of immune escape in triple-negative breast cancer (TNBC), offering a novel perspective for the enhancement of cancer immunotherapy. A separate study demonstrated that BCa cells hijack the neuron-astrocyte metabolic coupling to promote brain metastasis by secreting miR-199b-5p [24]. This suggests a potential target for therapeutic strategies against brain metastases in BCa. Therefore, microRNAs are a promising and safe biomarker for disease diagnosis and treatment, offering significant potential for personalized medicine. Currently, there is a lack of relevant studies both domestically and internationally confirming the associations of miR-1911-3p, miR-4694-5p, miR-548ao-5p, and miR-4804-3p with the development of BCa. This study validated significant reductions in the serum levels of miR-1911-3p, miR-4694-5p, miR-548ao-5p, and miR-4804-3p in BCa patients through three-phase testing, suggesting that these miRNAs may play an important role in the occurrence and development of breast cancer. Several studies indicate that a molecular signature, combining multiple biomarkers, provides greater specificity and accuracy for diagnostics than individual biomarkers [25]. Hong’s experiment established a panel of eight miRNAs (miR-324-5p, miR-10b-5p, miR-455-3p, miR-486-5p, miR-20a-5p, miR-107, miR-146b-5p and miR-139-5p) to predict recurrence in TNBC patients and offer personalized treatment [26]. This study identified miR-548ao-5p and miR-4804-3p as independent inhibitors of BCa through multivariate analysis. Their expression patterns were consistent in both serum and tissues of BCa patients. However, no significant differences were found in the levels of these microRNAs among hepatocellular carcinoma, gastric cancer, and lung cancer patients. These findings suggest that miR-548ao-5p and miR-4804-3p may serve as non-invasive biomarkers for BCa diagnosis, providing a promising alternative to traditional tissue biopsy methods.

Serum tumor marker testing is widely utilized in clinical laboratories as an indicator because of its convenience, low invasiveness, and cost-effectiveness in clinical development [27]. The most commonly used serum tumor markers in BCa screening and auxiliary diagnosis include AFP, CEA, CA153, CA152, and CA199. However, a single tumor marker is usually insufficient for accurate diagnosis. The use of multiple markers can increase the accuracy of diagnosis, and different combinations of markers yield varying diagnostic values [28]. Luo’s research confirmed that among the various combinations of three parallel detections of BCa tumor markers, the sensitivity of AFP + CEA + CA153 was the highest, whereas the AUC of CEA + CA125 + CA199 was the highest [29]. Our study also revealed that both CA125 and CA153 independently contribute to breast cancer incidence, but they both exhibited poor sensitivity. However, when these methods were combined, they had greater higher diagnostic effectiveness. Although numerous studies have demonstrated that molecular signatures composed of multiple miRNAs are valuable for the diagnosis and treatment of BCa, the diagnostic utility of miRNAs combined with serum tumor markers remains poorly understood. (miRNA + TM) Panel III, a novel diagnostic model of miRNA combined with tumor markers, was developed. This approach addresses the issue of low sensitivity associated with serum tumor markers and demonstrates excellent sensitivity, specificity, accuracy, stability, and overall diagnostic performance. The robustness of this model was validated through multi-phase design and comprehensive comparative analyses from various perspectives. This finding addresses a significant gap in BCa research regarding novel miRNAs, which not only has substantial scientific merit but also holds potential for breakthroughs in diagnosis and treatment.

Breast cancer is a highly heterogeneous malignancy, with diverse tumors in different patients in terms of growth rate, aggressiveness, hormone dependence, and treatment response [30, 31]. ER, PR and HER2 are widely used indicators for targeted BCa treatment and prognosis assessment [32, 33]. Invasive breast cancer can be categorized into four major molecular subtypes, namely, luminal A, luminal B, HER2-enriched, and triple-negative subtypes, which are based on hormone receptor status and gene expression patterns [34]. The management of BCa is complex and involves personalized treatment tailored to the molecular subtypes of patients. Scholars have proposed that miRNAs may play a role in regulating the pathogenicity of tumors by disrupting signaling pathways, potentially influencing the response to hormone therapy in the luminal A and luminal B subtypes of BCa [35]. For example, high expression levels of miR-221/222 promote the transition of BCa cells from ER-positive to ER-negative, while miR-221/222 sponge can effectively treat Tamoxifen-resistant BCa patients through G1 cell cycle arrest and restoration of ER-α expression [36]. Multifactorial linear regression revealed a strong correlation between miR-548ao-5p and miR-4804-3p concerning HER2 status. Patients with HER2-enriched breast cancer often receive targeted anti-HER2 therapy, which is linked to an aggressive clinical course and poor prognosis [37]. As clinical trials advance, an increasing amount of research is focusing on targeted delivery systems. These include the use of engineered cellular carriers [38], self-assembling nanoparticles [39] and photo-responsive cationic liposome platform [23] to deliver microRNAs to specific sites for therapeutic purposes. Our study shows that miR-548ao-5p and miR-4804-3p are linked to the onset and progression of BCa. In the future, combining targeted therapies with microRNAs is expected to improve treatment efficacy for HER2-positive breast cancer, leading to more precise personalized treatment strategies.

The mortality rate of BCa has decreased in recent years. Approximately 20–30% of patients are estimated to experience metastasis after diagnosis or treatment [40]. The prognosis of invasive breast cancer is significantly impacted by the tumor-node-metastasis (TNM) staging system [41]. TNM anatomic staging predicts the overall stage of the disease by combining the quantitative classification of primary tumors (T), regional lymph nodes (N), and distant metastases (M) [42, 43]. A lower histologic grade, positive PR, and fewer positive lymph nodes are independently correlated with a low-risk recurrence score [44]. Currently, anatomic staging is combined with prognostic staging to provide a more comprehensive diagnosis, prognosis, and cancer management. Studies have indicated that patients with a larger tumor diameter or lymphatic proliferation are associated with an increased rate of early mortality [45]. Du et al. reported that the expression of miR-92b-3p increases in BCa patients with larger tumor diameters, low tumor differentiation, high TNM stage, and lymphatic metastasis, which suggests that miR-92b-3p could serve as a potential biomarker for both the diagnosis and prognosis of BCa [46]. Additionally, the downregulation of miR-150-5p is closely linked to larger tumors, higher p53 expression, increased tumor recurrence, the presence of distant metastasis, and poor survival outcomes [47]. The 5-year survival rate for patients with stage I significantly exceeds that of patients with distant metastases [48]. This study revealed a correlation between the expression of miR-548ao-5p and miR-4804-3p and the TNM anatomic staging of BCa. These was a significant decrease in the serum levels of these microRNAs in patients with stage III/IV disease as well as those who had undergone lymph node transplantation, with a decreasing trend noted as the T segmentation level increased. These findings clearly indicate that miR-548ao-5p and miR-4804-3p can serve as indicators for the progression of BCa and are closely associated with clinical prognosis.

In this study, we used the TargetScan, miRDB, and miRWalk databases to predict the target genes of miR-548ao-5p and miR-4804-3p. Target gene identification was performed using the Gene Expression Profiling Interactive Analysis (GEPIA) database, The University of ALabama at Birmingham CANcer data analysis Portal (UALCAN) database, Kyoto Encyclopedia of Genes and Genomes (KEGG) pathway analysis, and Gene Ontology (GO) enrichment analysis. Our findings indicate that miR-548ao-5p may regulate cyclinB1 expression by targeting Heterogeneous Nuclear Ribonucleoprotein F (HNRNPF), thus inhibiting cell cycle progression and acting as a negative regulator in breast cancer (data not shown). In addition, the candidate target gene functions of miR-4804-3p were primarily shown to regulate DNA-binding transcription factors and ATF4-activated genes in response to endoplasmic reticulum stress (data not shown). Although this study’s robustness is enhanced by a multi-stage approach, it has several limitations. First, subjects were exclusively drawn from the First Affiliated Hospital of Guangxi Medical University and the Cancer Hospital of Guangxi Medical University, introducing regional constraints. Second, there is a lack of independent external validation and long-term survival data. In the future, we will conduct multi-center studies with hospitals from other regions to establish independent external validation, demonstrating the robustness of our diagnostic model across diverse populations. Additionally, we will incorporate more clinical variables for analysis and investigate the mechanisms of miRNAs to explore the potential of miR-548ao-5p and miR-4804-3p as biomarkers of BCa. In recent years, the investigation of miRNA combination diagnosis has transitioned from reliance on a single biomarker to an integrative approach that encompasses multiple omics disciplines, including radiology [49]. This field is advancing from laboratory settings into clinical applications. However, several challenges remain: first, there is an urgent need for large-scale clinical trial validation; second, standardizing data from various research platforms and sample sources is still unresolved. With advancements in artificial intelligence, nanotechnology, and body fluid biopsy techniques, miRNAs are expected to play a crucial role in early disease screening and personalized medicine in the future.

In summary, this study evaluated the diagnostic value of miR-1911-3p, miR-4694-5p, miR-548ao-5p, and miR-4804-3p in serum for BCa. A combined diagnostic model (including miR-548ao-5p, miR-4804-3p, CA125 and CA153) with high sensitivity, specificity, accuracy, and stability was successfully developed. This model overcomes the limitations of the low specificity of miRNAs and the low sensitivity of serum markers. The combined model shows very promising diagnostic performance and improved sensitivity for the early detection of BCa. Additionally, quantitative changes in miR-548ao-5p and miR-4804-3p may be associated with the progression and prognosis of BCa.

## Electronic supplementary material

Below is the link to the electronic supplementary material.

Supplementary Material 1

## Data Availability

No datasets were generated or analysed during the current study.
