# Supplementary material for: The diagnostic value of a breast cancer diagnosis model based on serum MiRNAs and serum tumor markers
Source: World J Surg Oncol. 2025 Mar 29;23:109. doi: 10.1186/s12957-025-03719-z (PMC11954258; doi:10.1186/s12957-025-03719-z)
Supplement: Supplementary file 1 — Supplementary Material 1 [file 12957_2025_3719_MOESM1_ESM.docx]

**Supplementary Materials**

**Supplementary methods**.High-Throughput Sequencing for the Screening of miRNAs.

**Supplementary table S1.** Validation of reference gene stability in qRT-PCR.

**Supplementary table S2.** Four miRNAs in the training phase were included in the logistic regression equation

**Supplementary table S3.** Serum tumor markers in the training phase were included in the logistic regression equation.

**Supplementary table S4.** The combination of miRNAs and serum tumor markers was included in the logistic regression equation during the training phase.

**Supplementary table S5.** Analysis of the correlation between the expression of miR-548ao-5p and miR-4804-3p and the clinical characteristics of patients with breast cancer

**Supplementary table S6.** Multiple linear regression analysis of the effects of ER, PR, and HER2 status on miR-548ao-5p.

**Supplementary table S7.** Multiple linear regression analysis of the effects of ER, PR, and HER2 status on miR-4804-3p.

**Supplementary figure S1.** Expression of serum markers in breast cancer patients and healthy controls.

**Supplementary method**s. High-Throughput Sequencing for the Screening of miRNAs

MiRNAs were extracted from serum exosomes of 4 breast cancer patients and 4 healthy controls. In order to eliminate the biological differences caused by different gene expression levels between samples, the serum exosome mirnas of the two groups were combined separately. Take 5 μL of the mixed sample as the initial volume of the library, and dilute the 3'-adapters and 5'-adapters at a ratio of 1:5. After adapter ligation, reverse transcription was performed, and purified cDNA libraries were generated through magnetic bead elution before amplification. The cDNA concentration was quantified using Qubit, while Qsep100 assessed its quality. The sequencing data was processed with Cutadapt software and reads of more than 17 nucleotides were retained for subsequent comparison with the miRBase database. The thresholds for differential expression of genes were set as |log2 (Fold Change)| > 5 and -log10 (PValue)≥2. Finally, candidate microRNAs for further experimental investigation were selected based on a review of tumor research literature.

|  |
| --- |

**Supplementary table S1.** Validation of reference gene stability in qRT-PCR

| variable | Range of Ct value | Minimum Ct value | Maximum Ct value | Mean Ct value ± SD | | P |
| --- | --- | --- | --- | --- | --- | --- |
|  |  |  |  | BCa | HC |  |
| U6 | 1.35 | 28.35 | 29.70 | 29.15 ± 0.39 | 28.96 ± 0.43 | 0.495 |
| Hsa-miR-16-5p | 2.91 | 25.15 | 28.06 | 26.49 ± 1.29 | 26.70 ± 0.83 | 0.755 |
| Cel-miR-39 | 1.86 | 17.37 | 19.23 | 18.14 ± 0.61 | 18.27 ± 0.70 | 0.785 |

|  |
| --- |

Abbreviations: Ct, Cycle Threshold

**Supplementary table S2.** Four miRNAs in the training phase were included in the logistic regression equation

| Variables | B | Standard Error | Wald | Significance | Exp(B) |
| --- | --- | --- | --- | --- | --- |
| miR-548ao-5p | -1.568 | 0.519 | 9.131 | 0.003 | 0.208 |
| miR-4804-3p | -1.134 | 0.51 | 4.935 | 0.026 | 0.322 |
| Constant | 2.494 | 0.397 | 39.55 | 0.000 | 12.108 |
| miR-1911-3p | 0.592 | 0.511 | 1.344 | 0.246 | 1.808 |
| miR-4694-5p | -0.152 | 0.686 | 0.049 | 0.825 | 0.859 |

**Supplementary table S3.** Serum tumor markers in the training phase were included in the logistic regression equation

| Variables | B | Standard Error | Wald | Significance | Exp(B) |
| --- | --- | --- | --- | --- | --- |
| CA125 | 0.150 | 0.037 | 16.066 | 0.000 | 1.162 |
| CA153 | 0.101 | 0.029 | 11.984 | 0.001 | 1.106 |
| Constant | -2.700 | 0.397 | 20.437 | 0.000 | 0.067 |
| CEA | 0.228 | 0.167 | 1.865 | 0.172 | 1.172 |

Abbreviations: CEA, carcinoembryonic antigen; CA125, carbohydrate antigen 125; CA153, carbohydrate antigen 153

**Supplementary table S4.** The combination of miRNAs and serum tumor markers was included in the logistic regression equation during the training phase

| Variables | B | Standard Error | Wald | Significance | Exp(B) |
| --- | --- | --- | --- | --- | --- |
| miR-548ao-5p | -1.173 | 0.527 | 4.947 | 0.026 | 0.310 |
| miR-4804-3p | -1.185 | 0.584 | 4.124 | 0.042 | 0.306 |
| CA125 | 0.123 | 0.041 | 9.116 | 0.003 | 1.131 |
| CA153 | 0.069 | 0.031 | 4.802 | 0.028 | 1.071 |
| Constant | -0.236 | 0.811 | 0.085 | 0.771 | 0.790 |

|  |
| --- |
|  |

Abbreviations: CA125, carbohydrate antigen 125; CA153, carbohydrate antigen 153;

**Supplementary table S5.** Analysis of the correlation between the expression of miR-548ao-5p and miR-4804-3p and the clinical characteristics of patients with breast cancer

| Variable | N | miR-548ao-5p expression | P | miR-4804-3p expression | P |
| --- | --- | --- | --- | --- | --- |
| Molecular Subtype |  |  | 0.003 |  | 0.021 |
| Luminal A | 25 | 0.68 (0.61) |  | 0.56 (0.66) |  |
| Luminal B | 42 | 0.44 (0.53) |  | 0.46 (0.54) |  |
| HER2-enriched | 21 | 0.24 (0.22) |  | 0.24 (0.22) |  |
| TNBC | 16 | 0.53 (0.70) |  | 0.56 (0.69) |  |
| ER status |  |  | 0.042 |  | 0.044 |
| Negative | 38 | 0.28 (0.39) |  | 0.28 (0.44) |  |
| Positive | 66 | 0.52 (0.54) |  | 0.52 (0.57) |  |
| PR status |  |  | 0.016 |  | 0.048 |
| Negative | 51 | 0.30 (0.44) |  | 0.35 (0.51) |  |
| Positive | 54 | 0.56 (0.52) |  | 0.50 (0.59) |  |
| HER2 status |  |  | 0.037 |  | 0.037 |
| Negative | 57 | 0.55 (0.59) |  | 0.54 (0.61) |  |
| Positive | 47 | 0.29 (0.42) |  | 0.32 (0.43) |  |
| TNM Classification |  |  | 0.003 |  | 0.043 |
| Stage Ⅰ/Ⅱ | 67 | 0.60 (0.56) |  | 0.50 (0.74) |  |
| Stage Ⅲ/Ⅳ | 47 | 0.31 (0.40) |  | 0.41 (0.38) |  |
| T |  |  | 0.003 |  | 0.012 |
| T1 | 34 | 0.68 (0.64) |  | 0.61 (0.86) |  |
| T2 | 52 | 0.49 (0.56) |  | 0.52 (0.53) |  |
| T3 | 16 | 0.43 (0.56) |  | 0.42 (0.43) |  |
| T4 | 10 | 0.17 (0.16) |  | 0.19 (0.17) |  |
| Lymph node metastasis |  |  | 0.034 |  | 0.012 |
| No | 54 | 0.60 (0.50) |  | 0.58 (0.67) |  |
| Yes | 58 | 0.35 (0.57) |  | 0.39 (0.45) |  |
| Distal metastasis |  |  | 0.066 |  | 0.218 |
| No | 91 | 0.55 (0.59) |  | 0.52 (0.63) |  |
| Yes | 24 | 0.32 (0.40) |  | 0.42 (0.37) |  |
| AFP (0.89-8.78) ng/mL |  |  | 0.692 |  | 0.792 |
| <2.83 | 60 | 0.52 (0.50) |  | 0.47 (0.53) |  |
| ≥2.83 | 61 | 0.48 (0.69) |  | 0.51 (0.60) |  |
| CEA (0-5) ng/mL |  |  | 0.119 |  | 0.113 |
| <1.83 | 60 | 0.47 (0.49) |  | 0.41 (0.53) |  |
| ≥1.83 | 61 | 0.56 (0.69) |  | 0.54 (0.60) |  |
| CA125 (0-35) U/mL |  |  | 0.043 |  | 0.036 |
| <13.5 | 60 | 0.60 (0.68) |  | 0.58 (0.68) |  |
| ≥13.5 | 61 | 0.42 (0.54) |  | 0.42 (0.50) |  |
| CA153 (0-31.3) U/mL |  |  | 0.586 |  | 0.608 |
| <15.2 | 60 | 0.56 (0.53) |  | 0.53 (0.59) |  |
| ≥15.2 | 61 | 0.48 (0.60) |  | 0.44 (0.48) |  |
| CA199 (0-37) U/mL |  |  | 0.446 |  | 0.502 |
| <8.06 | 60 | 0.51 (0.54) |  | 0.53 (0.50) |  |
| ≥8.06 | 61 | 0.39 (0.59) |  | 0.44 (0.62) |  |

Abbreviations: HER2, human epidermal growth factor receptor 2; TNBC,triple-negative breast cancer; ER, estrogen receptor; PR, progesterone receptor; TNM, tumor-node-metastasis; AFP, alpha-fetoprotein; CEA, carcinoembryonic antigen; CA125, carbohydrate antigen 125; CA153, carbohydrate antigen 153; CA199, carbohydrate antigen 199. The data in the aforementioned groups do not follow a normal distribution. The Mann-Whitney U test was employed to perform statistical analysis on two independent samples, while the Kruskal-Wallis H tests with Dunn's multiple comparison post-test was utilized for three or more independent samples. A two-tailed p-value of less than 0.05 was deemed statistically significant.

**Supplementary table S6.** Multiple linear regression analysis of the effects of ER, PR, and HER2 status on miR-548ao-5p

| Variable | Unstandardized Coefficients | | Standardized Coefficients Beta | t | Significance |
| --- | --- | --- | --- | --- | --- |
|  | B | Std. Error |  |  |  |
| Constant | 0.515 | 0.080 |  | 6.401 | 0.000 |
| ER+ vs ER- | 0.054 | 0.117 | 0.064 | 0.459 | 0.647 |
| PR+ vs PR- | 0.078 | 0.109 | 0.098 | 0.717 | 0.475 |
| HER2+ vs HER2- | -0.173 | 0.079 | -0.216 | -2.195 | 0.030 |

|  |
| --- |

Abbreviations: ER, estrogen receptor; PR, progesterone receptor; HER2, human epidermal growth factor receptor 2; ER-, PR- and HER2- were used as controls in this analysis.

**Supplementary table S7.** Multiple linear regression analysis of the effects of ER, PR, and HER2 status on miR-4804-3p

| Variable | Unstandardized Coefficients | | Standardized Coefficients Beta | t | Significance |
| --- | --- | --- | --- | --- | --- |
|  | B | Std. Error |  |  |  |
| Constant | 0.545 | 0.088 |  | 6.166 | 0.000 |
| ER+ vs ER- | 0.130 | 0.128 | 0.140 | 1.016 | 0.312 |
| PR+ vs PR- | 0.050 | 0.120 | 0.056 | 0.416 | 0.678 |
| HER2+ vs HER2- | -0.213 | 0.087 | -0.238 | -2.460 | 0.016 |

|  |
| --- |

Abbreviations: ER, estrogen receptor; PR, progesterone receptor; HER2, human epidermal growth factor receptor 2; ER-, PR- and HER2- were used as controls in this analysis.

**Supplementary figure S1.** Expression of serum markers in breast cancer patients and healthy controls


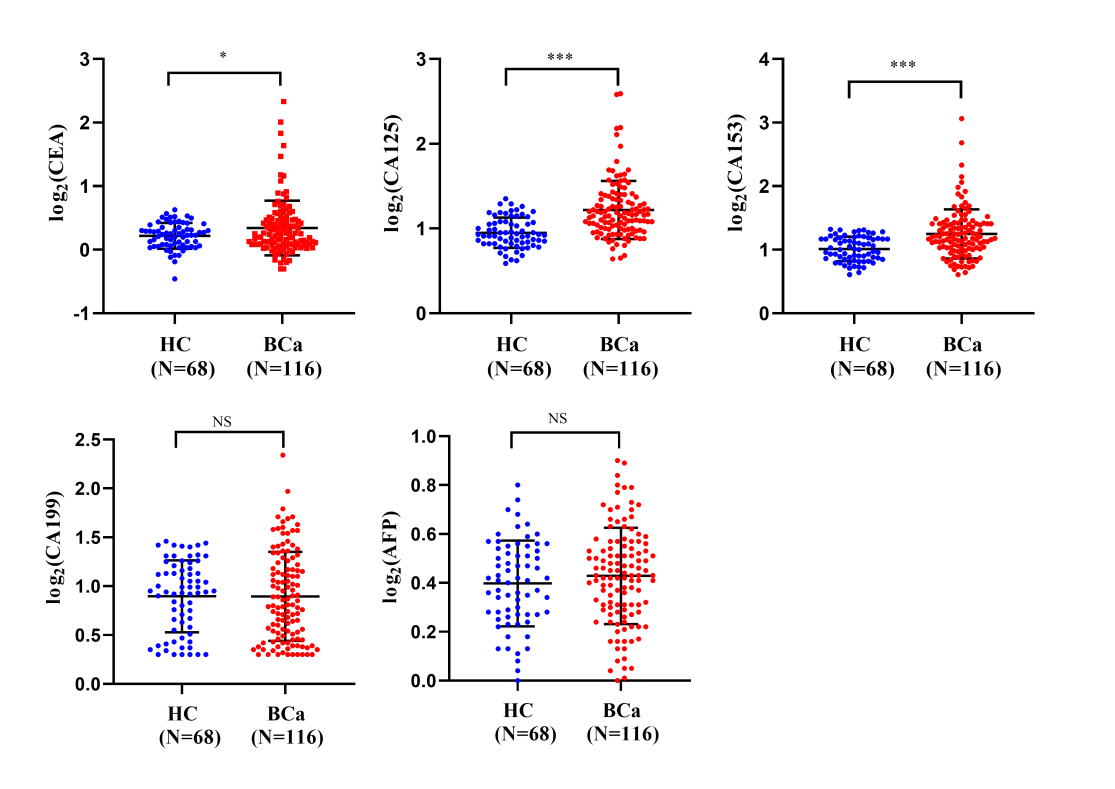


Abbreviations: AFP, alpha-fetoprotein; CEA, carcinoembryonic antigen; CA125, carbohydrate antigen; CA153, carbohydrate antigen 153; CA199, carbohydrate antigen 199. HC: Healthy control; BCa: Breast cancer;
